# ‘Mutiny on the Bounty’: the genetic history of Norfolk Island reveals extreme gender-biased admixture

**DOI:** 10.1186/s13323-015-0028-9

**Published:** 2015-09-03

**Authors:** Miles C. Benton, Shani Stuart, Claire Bellis, Donia Macartney-Coxson, David Eccles, Joanne E. Curran, Geoff Chambers, John Blangero, Rod A. Lea, Lyn R. Grffiths

**Affiliations:** Genomics Research Centre, Institute of Health and Biomedical Innovation, Queensland University of Technology, Q Block, 66 Musk Avenue, Kelvin Grove Campus, Brisbane, QLD 4001 Australia; Texas Biomedical Research Institute, San Antonio, TX 78227 USA; Kenepuru Science Centre, Institute of Environmental Science and Research, Wellington, 5240 New Zealand; School of Biological Sciences, Victoria University of Wellington, Wellington, 6140 New Zealand; South Texas Diabetes and Obesity Institute, University of Texas Rio Grande Valley School of Medicine, Brownsville, TX 78520 USA

**Keywords:** Norfolk Island, Mitochondria, Y-chromosome, Genetic isolate, Population history

## Abstract

**Background:**

The Pacific Oceania region was one of the last regions of the world to be settled via human migration. Here we outline a settlement of this region that has given rise to a uniquely admixed population. The current Norfolk Island population has arisen from a small number of founders with mixed Caucasian and Polynesian ancestry, descendants of a famous historical event. The ‘Mutiny on the Bounty’ has been told in history books, songs and the big screen, but recently this story can be portrayed through comprehensive molecular genetics. Written history details betrayal and murder leading to the founding of Pitcairn Island by European mutineers and the Polynesian women who left Tahiti with them. Investigation of detailed genealogical records supports historical accounts.

**Findings:**

Using genetics, we show distinct maternal Polynesian mitochondrial lineages in the present day population, as well as a European centric Y-chromosome phylogeny. These results comprehensively characterise the unique gender-biased admixture of this genetic isolate and further support the historical records relating to Norfolk Island.

**Conclusions:**

Our results significantly refine previous population genetic studies investigating Polynesian versus Caucasian diversity in the Norfolk Island population and add information that is beneficial to future disease and gene mapping studies.

**Electronic supplementary material:**

The online version of this article (doi:10.1186/s13323-015-0028-9) contains supplementary material, which is available to authorized users.

## Findings

### Historical background

**Fig. 1:**
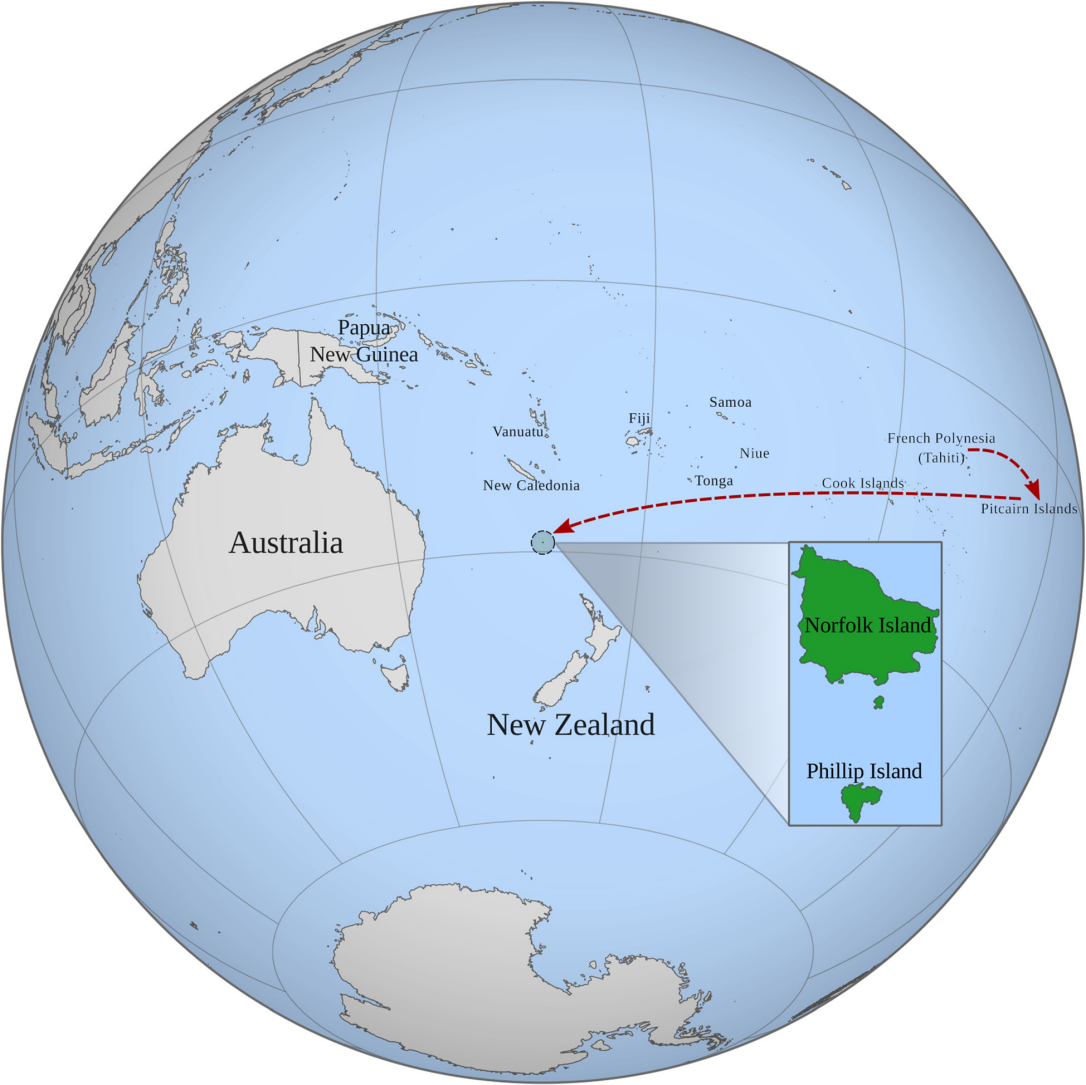
Orthographical map showing the location of Norfolk Island and surroundings. The movement of the European mutineers and Tahitian women to Pitcairn Island, and then Norfolk Island is indicated by the *dashed red line*. This figure was generated with custom perl scripts provided by in house collaborator David Eccles (script available at https://github.com/gringer/bioinfscripts/blob/master/perlshaper.pl)

Norfolk Island is located ~1400 km from the Australian East Coast (Fig. 1). This small island has an eventful history; initially populated and quickly abandoned by seafaring Polynesians, it was rediscovered in 1774 by Captain James Cook and used off and on as a British penal colony between 1788 and 1855 [1, 2]. Subsequently, the island became the home of a unique population, descendants of the mutiny of the Her Majesty’s Ship (HMS) Bounty.

The Bounty set for Tahiti in 1787, with the goal of securing and distributing breadfruit for the slave trade. After an unexpectedly long stopover in Tahiti, the majority of the Bounty crew overthrew Captain William Bligh. Led by Lieutenant Fletcher Christian, 18 mutineers returned to Tahiti. Expecting to be discovered by the British, Christian along with 8 other mutineers, 6 Tahitian men and 18 Tahitian women (plus a baby girl) fled to the Pitcairn Islands, where the Bounty was burnt to avoid detection and to eliminate the only viable escape route.

Conflict became rife, with numerous murders as well as suicide, leaving all Tahitian men and 7 mutineers dead. When Ned Young succumbed to respiratory failure in 1800, John Adams became the sole surviving male on Pitcairn. Slowly the island’s population grew, later bolstered by three further male European settlers—John Buffett and John Evans in 1823 and George Nobbs in 1828 [3]. These three men were the only outsiders to settle permanently on the island and subsequently married into the community.

Continual population growth and the concomitant pressure on resources led the British Government to allow the Pitcairn Islanders to settle Norfolk Island. On the 8th June 1856, a total of 193 inhabitants of Pitcairn Island were relocated. Today Norfolk Island has a population of about 1600 permanent residents, with the 2006 census reporting that approximately half of the permanent population are descendants of the Pitcairn founders [4].

Previously our group have illustrated the unique genomic properties of the Norfolk Island pedigree [5, 6], as well as its use in disease gene mapping studies [7–10]; establishing that this population isolate is able to identify both common and rare variants associated with complex phenotypes that may be difficult to study in larger but more heterogeneous populations. An important part of this process is identifying and understanding genetic admixture, it is critical to account for population substructure when conducting disease gene mapping studies. In this short report, we detail significant improvements to our previous estimates for both maternal and paternal ancestry in the unique population of Norfolk Island. These findings are interesting from a population genetics point of view and in turn highlight a key part of Oceanic history. Our results further comprehensively characterise the unique gender-biased admixture of this genetic isolate which is an important consideration when studying complex disease traits.

### Methods

#### Cohort collection and ethics

Accurate and detailed historical accounts have been used by genealogists to create and maintain a well-documented database of the entire Norfolk Island population, spanning all the way back to the original founders. This pedigree has been drawn up and is maintained in a genealogy program known as Brother’s Keeper. The pedigree includes ~5700 individuals coalescing over 11 generations or 200 years back to the original 9 European sailors and 12 Tahitian women. The Norfolk Island Health Study which has already been well established in previous research sampled individuals from the lower four generations of the pedigree and included 386 (64 %) individuals possessing lineages back to the founders and 216 individuals (36 %) who were considered to be new founders and did not show direct ancestral links. An updated core pedigree was constructed using this information and genetic information as it became available through genetic studies. Currently, the core pedigree structure contains those individuals that are most closely related and coalesce directly back to the original founders. The Norfolk Island Health Study (NIHS) has already been well established in previous research [7–10]. In this study, we used a representative group of Norfolk Island individuals selected from the pedigree, meaning that they relate back to the original founders, and we have phenotype and genotype information for them. The total number of core pedigree members selected was 330 (this was adjusted to exclude individuals under the age of 18 years), which consisted of 152 males and 178 females. All individuals gave written informed consent. Ethical approval was granted prior to the commencement of the study by the Griffith University Human Research Ethics Committee (ethical approval no: 1300000485) and the project was carried out in accordance with the relevant guidelines, which complied with the Helsinki Declaration for human research.

#### Orthographic map creation

A custom script was written by David Eccles to convert shapefile information into SVG images (https://github.com/gringer/bioinfscripts/blob/master/perlshaper.pl). This script was used with both 1:50 M resolution world political boundaries from NED (http://www.naturalearthdata.com/) and 1:1 M boundaries from GADM (http://www.gadm.org/) to create the local map and orthographic world projection in Fig. 1. Maps were combined together and overlaid with additional text using Inkscape (https://inkscape.org/en/).

#### Detection of the mitochondrial 9 bp repeat/deletion

To explore the presence of Polynesian mitochondrial genomes in the Norfolk Island population 23 individuals with direct maternal relationship to the Polynesian founders were selected. Primer sequences were designed to target the mitochondrial COII/tRNAlys region known to contain the 9 bp deletion in haplogroup B carriers; Forward primer 5′ AGGGCCCGTATTTACCCTATAG 3′, Reverse primer 5′ ATTTAGTTGGGGCATTTCACTG 3′. The target amplicon had an expected size of 133-bp for samples without the 9-bp deletion and 124-bp for samples containing the deletion. Briefly, 20 ng of genomic DNA was amplified with PCR Premix Optimisation Buffer E supplied by Epicentre Technologies, 0.3 μM of each mtDEL primer and AmpliTaq polymerase in a 20 μL reaction volume. Amplification was performed on a PE Applied Biosytems GeneAmp System 9700 with a 95 °C initial denaturation for 4 min followed by 30 cycles of 95 °C for 30 s, 60 °C for 1 min and 72 °C for 1 min, with a final extension at 72 °C for 7 min. Genotyping of the 9-bp deletion involved the use of fluorescently labelled primers. PCR products were incorporated into a formamide and ROX size standard mix prior to loading on an Applied Biosystems 310 Genetic Analyser. Fragment lengths were accurately determined after detection by automated gel capillary electrophoresis. Full details of the above method and RFLP data are available online: https://www120.secure.griffith.edu.au/rch/items/0e72bbad-feb0-c2bf-54d7-fef9adf7f5e3/1/.

#### Preliminary mitochondrial genome resequencing

Initially, the mitochondrial DNA of the selected 23 samples was resequenced with the VariantSEQr resequencing system (Applied Biosystems, Foster City, CA). The primer set (RS000056015_01 mitoALL) included a total of 46 amplicons. Each primer pair is tailed with universal M13 sequence and is designed for universal PCR and sequencing conditions. PCR was performed with a final volume of 10 μL containing 1.0 μL of genomic DNA, 2.0 μL mitoSEQr primer mix, 5.0 μL of 2X AmpliTaq Gold PCR Master Mix and 1.6 μL of 50 % UltraPure glycerol on a AB9700 with the following cycling conditions: Heat activation at 96 °C for 5 min, followed by 40 cycles of 94 °C for 30 s, 60 °C for 45 s and 72 °C for 45 s. A final extension of 72 °C for 10 min was included before cooling to hold at 4 °C. Prior to sequencing, PCR products were cleaned-up with 2 μL of ExoSAP-IT and 37 °C incubation for 30 min followed by heat inactivation at 80 °C for 15 min. A final volume of 10 μL contained 4.0 μL of BigDye Terminator Ready Reaction Mix v3.1, 1.0 μL of M13 primer, 3.0 μL of deionised water and 2.0 μL of PCR product. Cycling conditions were initiated with heat activation of 96 °C for 1 min, followed by 25 cycles of 96 °C for 10 s, 50 °C for 5 min and 60 °C for 4 min, a final hold step of 4 °C was included. To clean the sequence prior to electrophoresis, 2.5 μL of 125 mM EDTA was added to each tube and mixed followed by 30 μL 100 % ethanol. This solution was allowed to incubate at room temperature for 15 min. Following incubation, samples were centrifuged at 2000×*g* for 45 min with removal of supernatant prior to addition of 30 μL of 70 % ethanol and a further centrifugation of 15 min at 2000×*g*. Again, supernatant was removed, and samples were allowed to air dry before being resuspended in 10 μL of Hi-Di Formamide. Electrophoresis was performed on an AB3730xl (POP7) DNA Analyser running buffer with EDTA using a 36-cm array.

#### Sample preparation and complete mitochondrial genome sequencing

To facilitate more accurate and high throughput sequencing of complete mitochondrial genomes, we developed an in house method using a combination of long-range PCR and the Life Technologies Ion Torrent Next Generation Sequencing platform. This was performed on a representative cross section of the Norfolk Island pedigree (*n* = 322).

Mitochondrial DNA was enriched and purified before undergoing library preparation. Samples were amplified by long-range PCR, utilising two primer pairs which produce overlapping fragments covering the entire human mitochondrial genome. Primer sequences used were as follows: fragment 1 from 569 to 9819 b.p fwrd AAC CAA ACC CCA AAG ACA CC and rvrs GCC AAT AAT GAC GTG AAG TCC fragment 2 from 9611 to 626 b.p fwrd TCC CAC TCC TAA ACA CAT CC and rvrs TTT ATG GGG TGA TGT GAG CC. Primer sequences are given in the 5′ to 3′ orientation. Final reagent concentrations used were 1x Buffer, 0.5 mM dNTPs, 200 nM each primer, 3 % DMSO and 0.05 units/μL Roche expand long-range enzyme with 100 ng genomic DNA input in a 50 μL reaction volume. The optimum thermocycling conditions consisted of a two-stage PCR with an initial activation step at 92 °C for 2 min followed by 9 cycles of 92 °C for 10 s, 55 °C for 15 s, 68 °C for 9 min and 19 cycles of 92 °C for 15 s, 60 °C for 20 s, 68 °C for 9 min and a final extension of 7 min at 68 °C followed by an 8 °C hold. PCR products were run on 1 % agarose gels to accommodate for large fragment sizes at 70 V for 90 min and visualised under UV light. Ethidium bromide was used for staining at a 4 % concentration. Negative controls were included in each PCR run to check for contamination and ensure adequate quality control.

Mitochondrial PCR fragments were cleaned using QIAquick post PCR cleanup columns to remove any excess dNTPs or other reagents and were then quantified using Agilent DNA 12000 chips on a bioanalyser. Overlapping long-range PCR fragments were pooled together in equimolar amounts for each sample. After pooling and mixing 100 ng was aliquoted for library preparation. Samples underwent library preparation using NEBNext® Fast DNA Library Prep Set for Ion Torrent following the manufacturer’s protocol. Physical shearing using a Biorupter was found to produce a much more even distribution of fragments than an enzymatic approach. All samples were physically sheared post fragment pooling for 15 cycles (30 s ON, 30 s OFF) on high at 4 °C. Barcoding of samples was performed using Bioo Scientific NEXTflex DNA Barcodes for Ion PGM. The E-Gel SizeSelect system was used to select a target size of 350-bp libraries for use with 200-bp sequencing chemistry. Agencourt AMPure XP beads from Beckman and Coulter were used for all cleaning steps. After the final amplification and purification step all libraries were quantified on a Bioanalyser using DNA1000 chips. Samples for each multiplex were pooled in equimolar amounts and diluted to 26 pM, with 48 samples being plexed into a single sequencing reaction on 316 chips. Template preparation and sequencing was undertaken according to manufacturer’s instructions for the Ion Torrent platform.

#### Sequence analysis

A custom pipeline was developed to accommodate QC, alignment, assembly and analysis, with scripts available for download at a GitHub repository (https://github.com/gringer/phylotreescripts). Briefly, raw sequence reads were aligned to the Reconstructed Sapiens Reference Sequence (RSRS) [11] using Bowtie2 (http://bowtie-bio.sourceforge.net/bowtie2/index.shtml). SAMtools (http://www.htslib.org/) was used to produce Binary Alignment (BAM) files, which were processed into VCF files, then converted to consensus FASTA sequences. These FASTA sequences were compared with variant reference information from Phylotree in order to find mitochondrial haplotypes that most closely represented the consensus sequences for that individual.

#### Phylogenetic reconstruction

All phylogenetic reconstructions were generated in R (http://www.r-project.org/), using the APE package (http://cran.r-project.org/web/packages/ape/). Reconstruction and labelling of the Norfolk Island pedigree diagram was performed using the Kinship2 R package (http://cran.r-project.org/web/packages/kinship2/).

#### Y chromosome analysis

DNA extraction and genome-wide genotyping procedures for the NIHS are previously documented [10]. All SNPs located on the Y chromosome were QC filtered and extracted from genotype files using PLINK v1.07 (http://pngu.mgh.harvard.edu/~purcell/plink/); this is available as Additional file 1, Build36 map information is available as Additional file 2. The resultant SNP data were imported to R with haplotype and phylogenetic analysis performed using APE. SNP labelling was performed in accordance with the minimal skeleton Y tree [12].

### Results and discussion

#### Mitochondrial evidence for Polynesian female founders

**Fig. 2:**
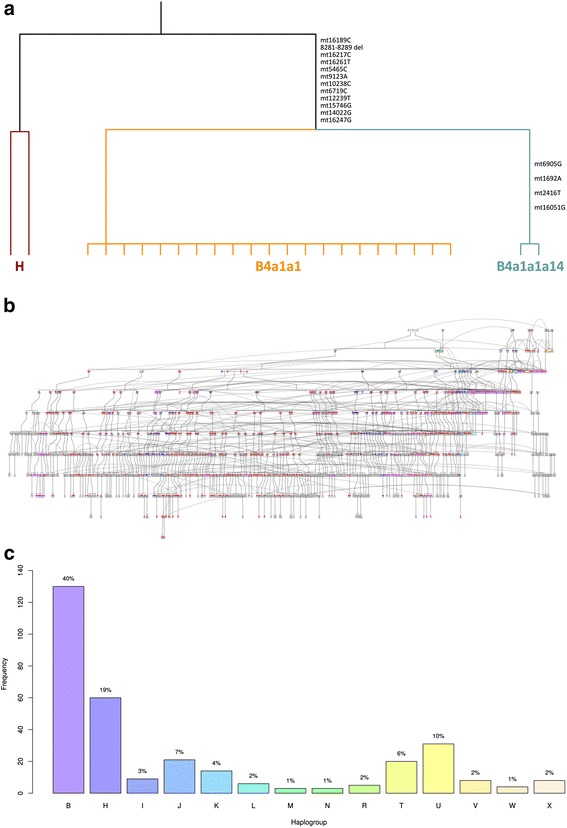
**a** Reconstruction of the mitochondrial phylogeny of 23 female Norfolk Island Polynesian founder descendants. The mitochondrial SNPs defining the branching are displayed on the tree. **b** Reconstruction of the Norfolk Island pedigree, based on available genealogical and genetic information (*n* = 1388). Four separate mitochondrial lineages have persisted and are present in the current day population (these are represented by *red*, *pink*, *blue* and *light blue* in the figure). **c** Major mitochondrial haplogroup frequencies as inferred from full mitochondrial genome sequencing in the Norfolk Island pedigree

Initially mitochondrial DNA (mtDNA) from 70 females believed to be related to the original founding population was investigated using a restriction-digest assay [13]. Detection of the COII/tRNA(lys) 9-bp deletion defines haplogroup B [14], this haplogroup accounts for >90 % of Polynesian mitochondrial haplogroups [15]. The genetic screen identified 23 individuals with the deletion, with genotypes being confirmed via mitochondrial resequencing (variant data is available as Additional file 3). This detected two specific Polynesian haplotypes within the current Norfolk population when aligned against the Cambridge Reference Sequence and another of European origin (haplogroup H, Fig. 2a). This tree shows two haplogroups; European (H; Cambridge Reference Sequence, and the investigator’s mtDNA) and Polynesian (B4). With the available variation, we were able to detect 2 distinct haplotypes within the 23 B4 individuals; B4a1a1 and B4a1a1a14 (the latter has not been previously identified and is labelled in accordance with the latest build of Phylotree [16]). Interestingly, we note that clade B4a1a1m in phylotree is defined by 1692, 2416 (and 151) [16]; however, the clade we report (B4a1a1a14) shares these variants as well as 6905, potentially suggesting dubious positioning of the clade defined by 1692. Building upon this baseline haplogroup information obtained from the 70 females studied, we used genealogical information and available genetic data (SNP [10], and above mtDNA) to inform the reconstruction of a pedigree of 1388 people with direct ancestral links to the Pitcairn founders. This is a 1388 member pedigree tracing back ~11 generations (200 years) to the European mutineers/Whalers and Polynesian women. Six maternal Polynesian founder lineages are highlighted, individuals expected to have these mitochondrial genomes are displayed in colour throughout the tree (Fig. 2b, high resolution available in Additional file 4). It is apparent that of the six observed founding Polynesian women, potentially four separate mitochondrial lineages have persisted and are present in the current day population. By tracing the maternal Polynesian founder line through this pedigree, we estimated that 45 % of present-day individuals had Polynesian mitochondrial genomes defined by haplogroup B4a1, with additional lineages most likely derived from more recent female founders. This observation is substantially higher than previous results from a small study undertaken by our group using just 7 mitochondrial SNPs [6], which estimated Polynesian mitochondrial lineages at 24 % in the population. We wished to validate and refine this picture at an increased resolution and therefore undertook complete mitochondrial genome sequencing of key members of the Norfolk Island pedigree. These results reveal excellent concordance with the above findings, with 40.4 % (130/322) of the total mitochondrial genomes sequenced belonging to haplogroup B4a1[x] (Fig. 2c).

#### Y-chromosome evidence for European male founders

**Fig. 3:**
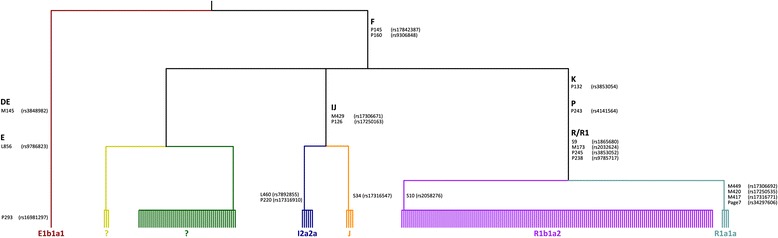
Y chromosome phylogeny of 223 male Norfolk Island individuals. Y-chromosomal SNPs were obtained from Illumina 610quad genotype data 12, 22 Y SNPs passed QC filters. Apart from one individual with haplotype E (defined by the P293 SNP; likely a recent founder), the remaining haplotypes are inferred to be of European origin. The Y chromosome SNPs defining the branching are displayed on the tree. There are 2 haplotypes within haplogroup F containing the P145 and P160 SNPs which we were unable to get enough resolution to accurately define, these are represented within the F branch and are coloured in *yellow* and *green*

Using 22 Y-chromosome SNPs, a phylogeny of Norfolk Island males (*N* = 223) was also constructed based on a minimal skeleton tree of Y haplogroups [12]. All but 1 male had Y-chromosome lineages derived from the haplogroup F-P145/P160 branch (for more detail see Fig. 3), indicating that they are almost certainly of European origin. While we were unable to completely resolve two haplotypes (indicated in green and yellow in Fig. 3) which contain haplogroup F-P145/P160 variants, these haplotypes have not been observed to date in Remote Oceanic populations [15], and therefore, in terms of current knowledge, are consistent with a non-polynesian heritage. However, it is possible that as more data becomes available for such Oceanic populations that these haplotypes may be observed. Y-chromosome lineages, like haplogroup G and haplogroup I1, which are common in North Europe along with haplogroup H (also common in India) occur elsewhere (e.g. South Asia) and are likely a consequence of maritime trade [17]. A single individual (haplotype E individual, P293, rs16981297) is associated with sub-clade E1b1a1. Not only does this isolated position in the tree compared to the majority of individuals studied suggest that this individual is a recent founder, but the fact that this sub-clade is commonly observed in sub-Saharan Africans [18, 19] provides further weight to this possibility. However, both further sample collection and analysis outside the scope of this project would be required to confirm the likelihood of this. This compelling evidence demonstrates that the vast majority of Y-chromosome lineages within the Norfolk Island pedigree are European in origin. This is consistent with previous work that typed just 4 common European Y-chromosomal SNPs [6]. The present study provides increased resolution of Norfolk Island Y-chromosome markers and supports the historical records associated with Pitcairn and subsequently Norfolk Island establishment.

#### Gender-biased admixture

The results presented here show excellent concordance with written historical accounts and greatly improve upon prior findings, refining estimates of sex-dependent admixture bias and specific haplotypic resolution within the Norfolk Island population. Sequencing of the mitochondrial genomes from a large majority of the pedigree members allowed us to ascertain that approximately 40 % of the current day population have Polynesian derived mtDNA. This is a large increase over our previous estimate of 24 % described in McEvoy et al. [6]. Similarly, we have been able to use information obtained from dense SNP arrays to infer the haplotypic status of 240 males based on Y-chromsome genotype data, which is a substantial improvement upon previous work that was limited to a small number of STRs. Overall, these new results add much more clarity to the genetic ancestral picture of the Norfolk Island population.

Knowledge of the population substructure caused by the divergence of genetically distinct ancestral populations is important when conducting disease gene mapping studies. For example, at the very least admixture requires adjustment when performing statistical tests of association within and between populations. The data presented here, unlike previous results, will allow accurate assessment and correction of sex-specific lineages when conducting disease gene mapping studies. In particular, when performing disease gene mapping studies accurate assignment of the mitochondrial haplogroups that distinguish between and within Polynesian and European ancestry subgroups should allow real pathological point variants in the mitochondrial genome to be discerned from those increasing in frequency due to population genetic factors such as genetic drift.

The geographic isolation of Norfolk Island, alongside a small number of European and Polynesian founders—including the additional 3 European men settling Pitcairn later—have established a unique genetic isolate. Consequently, extreme gender-biased admixture contributes to this unique genomic structure and provides exciting opportunities for future population genetics and disease gene mapping studies.

## Additional files

Additional file 1:
**List of Norfolk Island Y chromosome SNP genotypes.** This file contains Y chromosome genotypes of the Norfolk Island males reported in this study. (CSV 75 kb) Additional file 2:
**Y chromosome SNP map information.** This file contains the SNP map information for the Y chromosomes listed in the above file (Additional file 1). Note: map information is human genome Build36. (CSV 3 kb) Additional file 3:
**Mitochondrial variants from the initial resequencing assay.** This file contains variant data from the mitochondrial resequencing assay. (CSV 1 kb) Additional file 4:
**High resolution reconstruction of the 1388 member Norfolk Island core-pedigree.** This figure is provided to allow increased detail viewing of the reconstructed pedigree found in Fig. 2b. The colours each signify a mtDNA lineage from an original Polynesian maternal founder. (TIFF 2996 kb)

## Abbreviations

HMS: Her Majesty’s Ship
mtDNA: Mitochondrial DNA
NIHS: Norfolk Island Health Study
PCR: polymerase chain reaction
QC: quality control
RFLP: restriction fragment length polymorphism
SNP: Single Nucleotide Polymorphism
STR: short tandem repeat
