# Erratum to: ‘Mutiny on the Bounty’: the genetic history of Norfolk Island reveals extreme gender-biased admixture

**DOI:** 10.1186/s13323-015-0029-8

**Published:** 2015-10-07

**Authors:** Miles C. Benton, Shani Stuart, Claire Bellis, Donia Macartney-Coxson, David Eccles, Joanne E. Curran, Geoff Chambers, John Blangero, Rod A. Lea, Lyn R. Griffiths

**Affiliations:** Genomics Research Centre, Institute of Health and Biomedical Innovation, Queensland University of Technology, Q Block, 66 Musk Avenue, Kelvin Grove Campus, Brisbane, 4001 QLD Australia; Texas Biomedical Research Institute, San Antonio, 78227 TX USA; Kenepuru Science Centre, Institute of Environmental Science and Research, Wellington, 5240 New Zealand; School of Biological Sciences, Victoria University of Wellington, Wellington, 6140 New Zealand; South Texas Diabetes and Obesity Institute, University of Texas Rio Grande Valley School of Medicine, Brownsville, 78520 TX USA

Unfortunately, the original version of this article [1] contained an error. The spelling of the author name Lyn R. Grffiths was incorrect. The correct author name is Lyn R. Griffiths. The author name has been corrected in the original article and is also included correctly in author list above.
