# Supplementary figures and images for: ‘Mutiny on the Bounty’: the genetic history of Norfolk Island reveals extreme gender-biased admixture
Source: Investig Genet. 2015 Sep 3;6:11. doi: 10.1186/s13323-015-0028-9 (PMC4558825; doi:10.1186/s13323-015-0028-9)

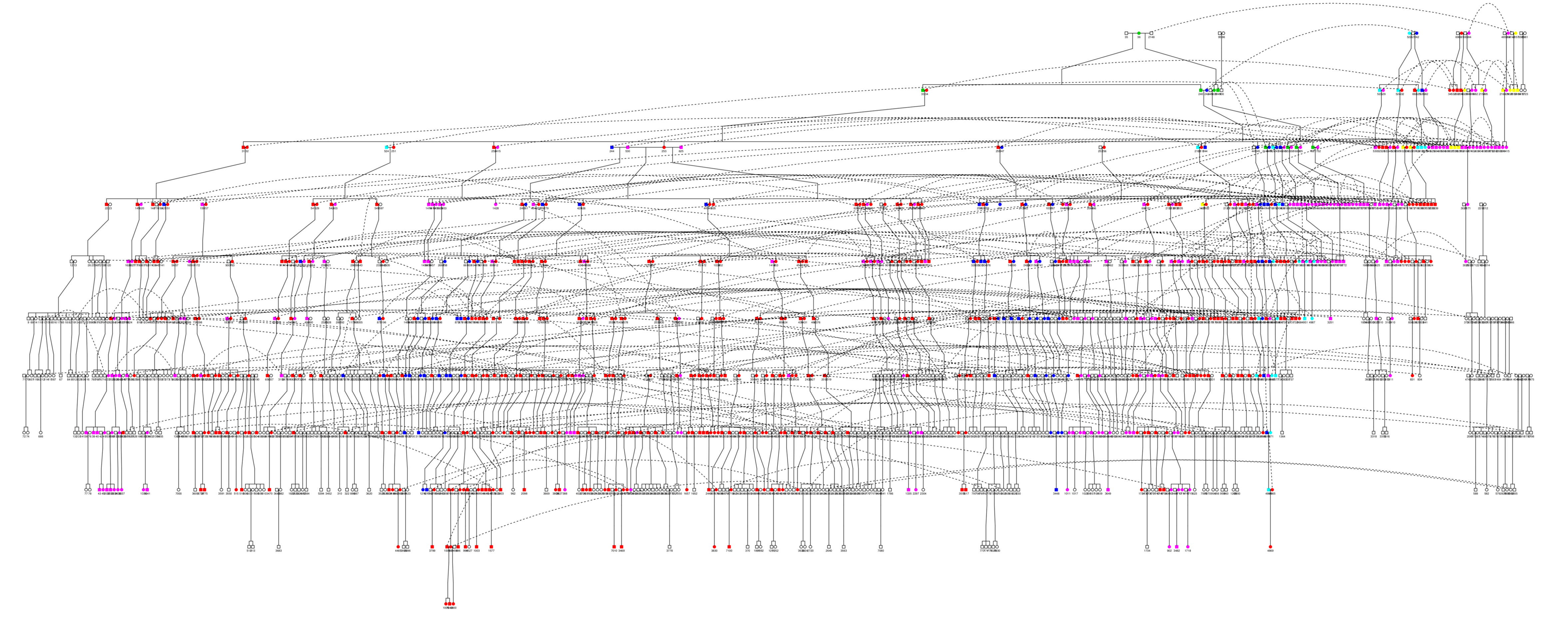

Supplement: Additional file 4: — High resolution reconstruction of the 1388 member Norfolk Island core-pedigree. This figure is provided to allow increased detail viewing of the reconstructed pedigree found in Fig. 2b. The colours each signify a mtDNA lineage from an original Polynesian maternal founder. (TIFF 2996 kb) [file 13323_2015_28_MOESM4_ESM.tiff]
